# Is There a Problem in the Laboratory?

**DOI:** 10.3389/fpsyg.2018.02443

**Published:** 2018-12-05

**Authors:** Alexander Nicolai Wendt

**Affiliations:** Department of Psychology, Universität Heidelberg, Heidelberg, Germany

**Keywords:** problem-solving, phenomenological psychology, field theory, demand characteristics, live streaming

## Abstract

Problem-solving research in the field of psychology has been closely linked to laboratory investigations throughout its development. However, there is a questionable conceptual assumption underlying this commitment to the laboratory, namely the assumption that one can reduce all problem-solving behavior to a cognitive mechanism. Upon validating this assumption from a phenomenological standpoint, doubts about its foundations emerge. For when we consider the experiential conditions that characterize a problematic situation, we come to determine several phenomenal aspects that are not taken into account in this approach. A phenomenologically revised notion of the problem therefore demands a modification of the scope of the empirical research. First, this paper investigates the configuration of the laboratory as an arena of experience based on Lewin’s field theory. This investigation indicates instructions as a key component of the laboratory. Second, a phenomenological description proposes a novel understanding of the problem. In this part it is shown that it is wrong to presume that problematic situations can be evoked arbitrarily by instructions. Finally, further contemplations help outlining the empirical requirements for exhaustive research. They call for novel paradigms in empirical psychology, such as live streaming, which are more faithful to the phenomenology of problems.

## Introduction

From an everyday point of view, problem-solving appears to be an entirely common occurrence. It seems as if the emergence of any kind of goal or urge brings with it the need for a corresponding solution. Whether selecting a menu for dinner or playing a friendly game of chess, participating in a marathon or working on a mathematical equation, the “goal-directedness” (Ohlsson, 2012) of these activities entails the idea of a solution – which is usually seen as the key feature of problems. This intuitive first look into everyday life favors agreeing with Popper’s notorious credo, “all life is problem-solving” (Popper, 1999).

But is the possibility of there being a solution or mere goal-directedness really sufficient to pose a problem in the first place? One cannot answer such a question from an everyday point of view alone, because this view is already linguistically committed to a vaguely holistic use of the word “problem.” Yet, everyday language indicates some excess of phenomenal meaning by talking about the “pressure” people feel in addressing a problem, the “trouble” caused by a problem, or the habit of “problematizing” something in order to defamiliarize common sense. When saying “I hope this is no problem,” it ought to be implicitly clear that there is no interest in formal goal-directedness but rather some emotional involvement. Equally, talking about a “huge problem” does not indicate an extraordinary goal but an issue of utmost relevance. These colloquialisms hint at a specificity of problems which does not concur with the global notion of “all life is problem-solving.”

For psychology as an empirical discipline, which has sought to investigate problem-solving in the tradition of the classical “human problem solving” approach by Newell and Simon (1972), there does not seem to be an immediate conflict from the ambivalence either. Firstly, the theoretical construct of problem-solving can be determined by a functionalist formula, such as Newell’s and Simon’s idea of the “task environment”: problem-solving here refers to the transformation of an initial state into a goal state by overcoming barriers (for a detailed discussion of the cognitivist paradigm of problem-solving research, see Wendt, 2017a). Secondly, this operationalization is investigated on the basis of empirical data. Thus, although it is unclear what a problem really is, empirical research can still investigate problem-solving since there is no such essential claim but rather the purpose to validate an abstract construct. This is the point where the investigation of constructs disconnects from actual experience, since investigation itself does not seek to contribute to the understanding of factual problems.

As long as the everyday conception of problems appeared to be the trivial “all life is problem-solving,” psychological problem-solving research did not have to justify its construct by drawing on a functionalist concept. In other words, while problems are taken to be self-evident occurrences, such psychological research intuitively appears to be relevant for the entirety of everyday life. Gazing into the phenomenal complexity of problems, however, not all life is problem-solving and there is no alternative concept that is ready to serve as an everyday frame for the psychological construct. Instead, only a thorough investigation of the experiential qualities of problems can determine the actual relevance of psychological investigations.

Various keen and skeptical psychologists have noticed this issue (such as Getzels, 1982; Quesada et al., 2005; Ohlsson, 2012; Funke, 2014). These researchers prefer the ambition of investigating actual experience over the mere elaboration of constructs. Dörner and Funke (2017) have recently highlighted the importance of recovering the original motivation of problem-solving research to be involved with “complexity and uncertainty in the world” (8). The existence of such a contribution shows that the scientific field of psychology cannot be represented accurately by means of a single methodological conviction. Rather, the discipline is characterized by a constitutive controversy which nurtures its progress.

So far, there has been little interest in the meaning of instructions, tasks, or demand effects for the experiential constitution of problems due to its ostensible ubiquitous nature (“all life is problem solving”). Phenomenological analysis, however, might be able to show that laboratories sometimes fail to provide peculiar requirements of experiencing problems. In light of such realization, the aims of problem-solving research can be readjusted: either the research interest favors “tasks” over “problems” as an adequate subject matter, or explorations into less obtrusive empirical methods, such as live streaming, are undertaken.

In the following sections, three steps are undertaken. In the first step (see section “Situations in the Lab”), the situation given to an experimental subject in the arena of the laboratory is examined. This section’s question is whether it is possible to experience a genuine problem in such a situation. In the second step (see section “The Phenomenology of the Problem”), these considerations lead to a phenomenology of the problem. It is asked which experiential qualities are paramount when having a problem. In the last step (see section “Empirical Approaches”), new opportunities to investigate such phenomenologically authentic problems will be explored.

## Situations in the Lab

The aim of the first step is to determine the experiential conditions that subjects are faced with when participating in a laboratory investigation. Psychological research has not been oblivious of such effects. On the contrary, various reflections on its own methodology yielded an advanced understanding of subjects’ behavior in laboratories. However, it is necessary to orientate these findings toward those types of situations which are presumed to be problems. The question is whether or not the task environment of laboratories actually hosts the opportunity for a subject to have a problem.

Experience is necessarily situated and having a problem is one way to be situated. Before exploring the nature of problematic situations, however, it is helpful to investigate the *status quo* of contemporary psychological research in the field of problem-solving, namely the laboratory as an arena, i.e., as a spatially arranged and therefore socially standardized situation. The status of the laboratory as the classical data source and site of investigation in experimental psychology has been subject to continuous skepticism due to an apparent lack of external validity (e.g., Anderson et al., 1999), especially when it comes to inferences made about other contexts from the observations that are made in the laboratory.

Yet, this controversy about the place of generalization as a structural equivalence between the laboratory and the rest of the field is not or merely indirectly relevant to the present matter. Moreover, this line of argument does not scrutinize the situational singularity of everyday life, since the notion of external validity supposes good laboratory research to be estimated in accordance with some prevalent normative set of rules. This idea can be easily refuted because it does not emancipate itself from the pre-phenomenological natural attitude. Instead, the basic understanding of the variety of situations which are applied here has to refuse any previous hierarchy.

In order to grasp the peculiarities of the laboratory, the description should include two approaches. First, there are several properties of the laboratory which are a concern for psychological methodology themselves, or specifically which are a concern for the systematical investigation of the psychological structure underlying the discipline’s data sources. By way of example, an important effect on the behavior of subjects has been labeled “demand characteristics” by Orne (1962). Orne in this study observed that the subjects’ actions, motivations, and perceptions about the experiment play an active role in the observed behavior. Throughout communication about the experimental situation, such as recruitment, the experimenter’s instructions or the laboratory’s setup, the subjects perceive “demand cues” which provoke a change of attitude toward the situation.

There are two ways to comprehend these characteristics (Sharpe and Whelton, 2016). Either they are “artifacts,” which means that one sees them as a contamination of an experiment’s reliability, that they can be present or absent in the laboratory, and that the researcher is able to eliminate them. Or they are a “discovery,” which means that they are elements of each and every laboratory’s situation. As they write, “as artifacts, demand characteristics are restricted to those subjects who can express the *experimenter’s* hypothesis. But, as a discovery, demand characteristics highlight the importance of ascertaining the *experimental* hypothesis as understood by the research subject” (Sharpe and Whelton, 2016, 360). If we accept the second view, for theoretical reasons developed in what follows, demand characteristics as a discovery do not entail the assumption that the experimental subjects try to understand the particular hypothesis of the experiment in which they are currently partaking. Instead, one can understand being part of an experiment as a state which influences the participant’s consciousness in general.

Moreover, the present descriptive attempt can subsume a different group of effects recognized by empirical psychology under the label “task instructions.” Di Mascio et al. (2016, 1) clarify this terminology as follows: “Task instructions may work by providing a motive for problem-solving. Organized cognition—such as that involved in problem-solving—is motivated. Motives are valued goals for the problem-solving activity that can guide the duration and direction of attentional effort.” Hence, different task instructions tend to cause different behavioral responses in the experimental situation even if the remaining setup is constant. This variety can be explained as a result of behavior’s context-dependency (Braem et al., 2017). There are various ways to produce effects of task instructions, such as wording, placement, or elaboration of the instruction.

In the further context of psychologically recognized occurrences in the laboratory situation, effects of compliance (Asch, 1951), social desirability (Crowne and Marlowe, 1960), experimenter expectancies (Rosenthal and Rubin, 1978), the Hawthorne effect (Landsberger, 1958) or framing (Tversky and Kahneman, 1981) all become relevant. Together they illuminate the remarkable impact of the laboratory as an arena or situation on the experimental subjects. However, as empirical effects, they do not demand any implications because they are merely indicating probabilities of occurrence. This is why it is necessary to integrate these empirical findings into the context of a second, theoretically framed inspection of the laboratory as an arena for investigation.

Such a framework, with its function of describing the situational peculiarities of the laboratory, is Lewin’s field theory. Without subscribing to his topological or vector-psychological attempts that explain behavior and experience *more geometrico*, utilizing the descriptive faculties of Lewin’s field theory can still provide a useful aid to explicating situations. As a driving motivation behind the field-theoretical approach, Lewin states: “The psychological environment has to be regarded functionally as a part of one interdependent field, the life-space, the other part of which is the person” (Lewin, 1939, 878). Based on this assumption, it is possible to explicate the laboratory in its genuinely situational features and how it brings about the empirical effects discovered in experimental psychology.

In his major work Lewin (1936), Lewin lays out a conceptualized framework by which to understand the totality of facts that determine the behavior of an individual at a certain moment as the “psychological life space” (12) of this individual. However, by leaving no room for any speculative entities, Lewin also concedes the ability to give a “complete representation of even one given situation,” since this “would presuppose the solution of all psychological problems” (82). Instead, Lewin provides a framework of empirical concepts which explains the relation between person and environment as the life space that constitutes the experienced situation. This framework successfully illuminates the decisive factors which characterize the laboratory situation.

Life space is constituted by the individual’s “life situation” as well as “momentary situation” (23) and “the specific problem with which we have to deal in a given case determines whether it is the life situation or the momentary situation which comes more strongly into the foreground” (24). However, the life space does not contain the physical, social or conceptual world as it is, but merely “to the extent and in the manner in which they affect the individual in his momentary state” (*ibid*.). Therefore, Lewin employs the terms “quasi-physical,” “quasi-social,” and “quasi-conceptual” facts even though “a change in the quasi-physical facts in the life space of the person is often the result of an objective change in the physical environment” (27). This contingency between the life space’s content and the physical and social occurrences as well as concepts which surround it provides a shift of perspective from the idea of the laboratory situation as one might conceptualize it from the methodological point of view to what laboratory situation means within the subject’s life space. This meaning depends on the situation’s content. Lewin argues:

“Nevertheless, the content is in no way irrelevant, but is of greatest importance for psychological dynamics. Whether, for instance, an actual goal refers to a present or a future event, whether this event is thought of as something that definitely exists, or as something that is only possible or highly improbable – all this forms an essential characteristic of a goal. Differences in time index and in existential characteristics of the content imply a qualitative difference in the psychological facts themselves, that is, they have formally the position of properties of the psychological facts.” (38).

Thus, the goal that a person has does not depend on its conceptualization in experimental design, but on what it means in the individual life space. This thought becomes clear when considering Lewin’s notion of “alien influences,” i.e., “influences from outside on the psychological life space” (70). The life space is embedded in a hull of influences that do not immediately determine the psychological behavior. Yet, these influences impact the boundary points of life space by e.g., connecting it to other regions. The instruction given in the laboratory is seen as such an “alien influence”:

“Every act of influencing another person, whether in laboratory experiment or in everyday life, consists in creating such a hull, one which affects the boundary points of the life space and thereby the life space itself in a certain way.” (75).

Hence, the critical point is the notion of experimental instruction, since this notion is not necessarily itself a quasi-social fact. Yet, it can create such a quasi-fact by its impact on the individual’s life sphere. Talking about the Zeigarnik effect, Lewin (1940, 19) states that “the instruction to recall given by the experimenter sets up a quasi-need.” This “quasi-need” is the possible impact of the instruction on the experimental subject’s life space. As he says, “it is able to impose certain patterns of action and to build up certain quasi needs” (Lewin, 1936, 192), but there is no identical representation of the instruction’s content.

At this point it is important to relate Lewin’s theoretical reflections to the psychological effects encountered in the arena of the laboratory, because the isolated notion of quasi-needs still allows for alternative meanings. Pointing toward the research interest of problem-solving investigations, the criterion needs to be that this quasi need serves to problematize the situation if the claim is that psychology investigates actual problem-solving. As its reference, however, any comment on this concern still requires the phenomenology of the problem.

Psychology’s task is to recover the problem in life space as its subject matter instead of trying to induce problems. This task neither implies an entire loss of the credibility of past research, nor does it encourage a divergence from established empirical paradigms. In any case, real problem-solving research needs to turn to properly address the subjective situational conditions of what it means to have a problem. For Lewin, therefore, the concern of valid empirical research is a matter of adequacy: “the validity of sociopsychological experiments should be judged not by the properties of isolated events or single individuals within the field but mainly by whether or not the properties of the social group or the social situation as a whole are adequately represented” (Lewin, 1939, 893). Concerning problem-solving, the “social situation as a whole” has to be read as a reference to the entire experience of a problem instead of as a formal and mechanistic definition of problems predominant in contemporary research.

The contingency between instruction and quasi need might constitute the most pronounced example of the influence of the laboratory situation on experience. It is this setup as a whole which occasions the change within life space. The reason for this change is that “experimentation in the laboratory occurs, socially speaking, on an island quite isolated from the life of society” (Lewin, 1944, 168). Such special status affects the individual in a peculiar way. It serves as the comparative instance to life space once this individual is involved in a problem.

Notwithstanding, one might wonder whether the Lewinean considerations about the state of the life space in the laboratory situation does coincide with the methodological considerations about external validity of empirical research in general. This might be true to a certain extent because, formally, the singularity of the laboratory setting queries all generalizing inferences. However, this formal relation is only a consequence of an underlying experiential structure. Such considerations concern what Lewin calls “Geschehnistypen” (types of event) in his earlier German writings (Lewin, 1927). When investigating the experience of problems, psychologists who worry about external validity alone would ignore the actual meaning of what a problem is.

Compared with this, the Lewinean starting point is independent from the distinction between field and laboratory. This means that the experience itself is initially examined without asking where it occurs empirically. If and only if the arena of experience affects the constitution of experience, there is a reason to analyze it. In other words, in other cases but problems, Lewinean field-theory might not necessarily stipulate doubts about the laboratory as the site of experience. In the light of such reflections, it becomes clear that thinking about the laboratory as a situation can only be relevant because it matters to the “Geschehnistyp” of a problem. Consequently, the issue of generalizability is derivative.

The first step has resulted in a critical view on the laboratory as a field of experience within the life space of experimental subjects. While its setup might be circumstantial for the investigation of other phenomena, it is for the peculiar nature of problems that the laboratory setup as a situation is conflated with peculiarities of this particular subject matter. Lewin’s field theory hosts a possibility of explaining how this conflation comes about: Tasks impose quasi-needs onto the subjects of investigation which inhibit the independent development of a genuine stance toward the situation. However, Lewin’s theory cannot account for the following step. Field-theory is committed to the naturalist view of New Positivism about external occurrences, a view which does not permit necessary phenomenological reflection on the experiential conditions of a problem.

## The Phenomenology of the Problem

Understanding that the artificial setup of the laboratory infringes the authentic development of a problematic situation provides the necessary premise for the second step, investigating the experiential qualities of authentic problems. The Lewinean analysis, as it were, renders the formal conditions of the problem as a situation in life space which stipulate a descriptive examination of the respective content. Therefore, the aim of the second step is to acquire an access to the experiential dimension of having a problem. Consequently, a shift from the outward perspective of field-theory to the inquiry of features which are intrinsic to the experience of problems is paramount.

To ask about the fundamental experiential qualities of a problem or a problematic situation ultimately calls for a phenomenological analysis. In his essay on phenomenology and psychology, Husserl (1917) pointed out that psychology relies on a naturalistic view of the phenomenon under question, while phenomenology seeks to inquire into the experiential conditions of such a view. This means that phenomenology does not investigate factual problems but rather the state of consciousness which establishes the experience of a problem. Inquiry into some of the features which qualify this experience have already been carried out in the history of phenomenological thought. Nevertheless, sketching out the phenomenology of the problem serves the purpose of demonstrating the complexity of experiential conditions involved in having a problem, as is required for a full critique of contemporary psychology.

Before obtaining the phenomenological attitude, the psychological tradition of thought about “human problem solving,” which has up until now only been cursorily elaborated, should be mentioned in passing. One can backtrack its historic development to the Gestaltist movement in the early 20th century (Goldstone and Pizlo, 2009), but the major development originates from the approaches in the 1960 and 1970s to computational simulation of problem-solving, especially by Newell and Simon (1972). This paradigm of cognitive psychology has remained dominant up until the 21st century and inspired various more specific studies in its wake.

However, it cannot pass unnoticed that cognitive sciences have experienced substantial paradigmatic shifts of their own since the emergence of theories of symbolic information-processing in the 1950s’ “cognitive revolution.” Thompson (2007, 4) distinguishes three major “approaches to the study of the mind,” namely “cognitivism, connectionism, and embodied dynamicism.” It is therefore questionable whether the earliest (phenomenological) critique directed against the classical cognitivist concepts (such as Dreyfus, 1972) still remains relevant to later and modern advances of experimental psychology.

What are the specifications of this cognitivist paradigm of “human problem solving,” which is to be criticized in the following section? First of all, it subscribes to the “Mechano-Representationalist Approach” (Hutto, 2008). The two predominant elements of this approach are, respectively, a mechanist understanding of the mind and a representationalist epistemology. Moreover, its explanations are functionalist, such as the infamous idea of the problem as a relation between an initial and a goal state. Ultimately, problem finding, problem classification, problem space, and problem quality (Wendt, 2017a) are the most pertinent research topics. Upon examination of some recent psychological contributions to this tradition of problem-solving research, it can be shown nonetheless that fundamental concerns still have not been overcome and resolved, despite critical advances in the last decades. Recent theoretical contributions to the field of problem-solving research certainly do address some flaws of prior theories, but they do not venture and lay out a genuinely new and totalist approach.

On the one hand, there is a growing number of attempts to advance cognitive architectures, such as Soar (Laird et al., 1987), ACT-R (Anderson, 1993), and Icarus (Langley and Trivedi, 2013). But despite dealing with conceptual problems, for example by integrating “depth first search” to compensate for the flaws of heuristic searches, or by treating “the physical side of problem solving as a central tenet” (Langley and Rogers, 2005) in order to establish a structure of embodiment, these attempts are still committed to mechanistic, externalist and representationalist premises which do not concur with the phenomenology of the problem (Wendt, 2017b).

On the other hand, there are more versatile approaches that reintegrate different perspectives from psychology. Weisberg (2015) elaborates on the relation between two psychological traditions of thought about insight as an impetus to problem-solving, namely the “special process” view following Gestaltist ideas and the “business-as-usual” view following cognitivism. Yet this controversial potential is located between two well-established psychological schools of thought which have equally been the object of extensive critique. Weisberg’s own proposal to integrate both views does not open up a new perspective, but accumulates the weak spots of its predecessors.

Similarly, with a return to concepts of Gestalt psychology, Yoshimi (2017) searches for a “phenomenology of problem-solving.” Still, approaches like this are mainly iterations of prior conceptual deficiencies. Nevertheless, Yoshimi’s idea to relate problem-solving to a “field of consciousness” draws attention to the fruitful tendency of shifting our reflective efforts toward the experiential conditions of the problematic situation at hand. The crucial phenomenological step to recover these conditions will thus be to acknowledge the situational autonomy of the problem, separating it analytically from the process and the action of solving.

But what does it really mean to say that I have a problem? First of all, there are two necessary conditions. Not all problems I face are my problems, even if they are problems that impact upon me. When talking about a “mathematical problem,” a “problem of chess,” or a “problem for science,” I do not necessarily have a problem myself. This initial consideration already clarifies an important difference which psychological problem-solving research ought to take into account. So In which cases is it justified to speak of having a problem? A problem is always a problem for someone. Facing a “mathematical problem” in a college exam, however, the task is considered a problem even before I know what the problem is. Arguably, it might be justified to speak of a problem in this case, because it will turn out to be a problem for every single student once it is presented to them, but there is also a custom to talk about “mathematical problems” even in their mere potentiality. This way one can speak of “mathematical problems nobody will ever discover.”

The difference between these problems and my problems is the experiential quality of mineness which should be distinguished from for-me-ness (Guillot, 2017). In the prior case, the problem entails a sense of ownership or intrinsic possession, whereas in the latter case there can also be an alien ownership. The imaginary “mathematical problem nobody will ever discover” belongs to an abstract and effectively empty third-person agency or, if anything, to mathematics: it is a problem for mathematics as a collective subject, rather than necessarily the problem of me or of any individual practicing mathematician. However, it would be inadequate to speak of mathematics (and any other impersonal entity) as if it had experiences. The use of the word “problem” to describe such cases is not literarily correct but rather stands as a metonym. To speak more precisely, it should be a “mathematical task,” a “configuration of chess,” or a “topic for science.” They might turn out to be someone’s problem, but they are not someone’s problem in the emphatic sense of the word before. Yet, when my problem actually is a “mathematical problem,” the adjective serves to qualify the domain of the problem. This ambivalence might be a temptation for psychologists to assume that any kind of so-called problem can make me have a problem, i.e., such that I stand in a problematic situation.

Another person’s problem can also be a problem for me whilst not being my problem. Playing a friendly game of chess with my niece, I can understand that the current situation is her problem, but the subjective character of mineness might still not apply for me. It can be a problem *for me* because I am conscious of the situation as a problem for someone else, but this does not mean that my experience of the situation can be seen as an authentic form of problem-solving since it is not *my* problem. Hence, mineness is a necessary condition for authentically experiencing a problem, but it is not a sufficient condition since the subjective character of mineness is also intrinsic to various other experiences. Nevertheless, the distinction between mineness and for-me-ness bears great utility in discarding situations that only appear to be my problems. It leads the way toward understanding the problem as a mode or “type of situation” (Dreyfus, 2004, 237) and, analogously, problem-solving as a form of what Dreyfus calls “skillful coping” in the tradition of the Heideggerian notion of “circumspection” (see for example Dreyfus and Dreyfus, 1988, 219). On the suggestion of phenomenology, empirical psychology should ultimately replace the ubiquitous assumption that “all life is problem-solving” with a subtler distinction, such as Dreyfus’ “skillful coping.”

Apart from instantiating mineness with a sense of ownership, my problem is a problem insofar as it is happening to me, is occurring to me, or is befalling me. In this sense, my problem affects me. Waldenfels (2015) utilizes the Greek term “pathos” to subsume these phenomena under a single semantic rubric. Such events display themselves in the form of eruptions, explosive noise, or sudden highlights of experience. The pathos of a situation is manifested in the affect by an alien object or the appeal or plea by another person. When a problem occurs, I am existentially exposed to this alterity.

Although it is my problem, it is neither constituted by a subjective act or cognition nor by an external process alone, but it is an event which occurs only insofar as it occurs *to* someone. The necessary conditions for problems of mineness and pathos intersect essentially at this point. The pathos of being affected by a problem can be experienced as anxiety, being puzzled, or even as paralysis, a symptom that resembles the experiences described by Gurwitsch: “somehow overwhelmed and overpowered by actual experience imposing itself upon them by a force of constraint from which they cannot emancipate themselves” (Gurwitsch, 1949, 179). Pathos is qualified by a peculiar temporal impression of being delayed: my problems occur because my solutions come too late, just as – in this sense – the occurrence of the problem came too early.

This delay reveals the second side to the phenomenon of pathos. Waldenfels adopts the term “responsivity” by Goldstein (1934) to describe that the occurrence inevitably demands a response. However, this response should not be understood in the tradition of behaviorist stimulus-response contingencies, but as an answer to affordances of pathos (Waldenfels, 1994). Moreover, responses are neither mere effects of causes nor results evoked by goal-directed behavior. Instead, situations are essentially open. This means that they demand a certain response but do not enforce it. In this field at the intersection of normality and anomaly, the subject’s responsibility emerges (in the sense of responsibility for the other, as proposed by Levinas, 1986).

Mineness, as a noetic quality of experience, and the dyad of pathos and response, are two distinct necessary conditions of problems. More specifically, the two necessary conditions of mineness and pathos-response provide a general situational frame which is ontologically connected with the questionability of life. With Sartre, this questionability can be anthropologically identified as “the question as a human attitude” (Sartre, 1956, 7) and bears an ontological relation to negativity: “Thus in posing a question, a certain negative element is introduced into the world” (23). Adopting a key formula by Fales, phenomenology can assess that “where there is no question there is no problem” (Fales, 1943, 69) but there are other, more peculiar, sufficient conditions to experiencing a problem.

This having been said, I do not have a problem to call my own just because I received a task or imagined a situation step by step. Rather, when I have a problem, I find myself in a predicament that requires my response because an existential change in my life is possible, although it is not inevitable, as it would be in the case of a catastrophe. To give a response to my problem is my responsibility, but this is equally true for opportunities, challenges, and fatalities. While mineness, pathos, and response should be seen as characteristics of certain situations of experience, the peculiar phenomenal features of problems are more specific.

The situation I encounter is essentially problematic because it is just as essentially solvable. This first sufficient condition of a problem does not entail the existence of a solution, nor the comprehension of one’s own goals. The functionalist idea that the goal state has to be given to perform problem-solving is equivalent to the “conviction that the commonsense knowledge problem must be solvable, since human beings have obviously solved it” (Dreyfus and Dreyfus, 1988, 223). Instead, the solvability of a problem resides in the feasibility to release the pressure inherent to the problem by means of a solution. When I have a problem, I approach what is happening to me “as if” (Vaihinger, 1965) there were a solution, as a matter of fiction. It is possible that I experience solvability where there is no known solution. While on the other side, a situation, despite having an otherwise known solution, might as well be experienced as a fatality – instead of a problem – since I do not find it solvable.

This fictional aspect of the experience of a problem relates to the possibility of “detach[ing] […] from the given situation and look[ing] at the latter from a distance” (Gurwitsch, 1949, 180). Gurwitsch calls this ability “thematization” in a Husserlian tradition, “meaning hereby disengagement and disclosure of factors which previously to the operation in question are present to consciousness in a rather implicit form” (*ibid*., 187f.). Still, this fictional characteristic of solvability does not entail a notion of illusion. Solvability, however, originates from my ability to hope that the problem can be solved.

Following Marcel, hope should be understood as “constituting our being’s veritable response” (Marcel, 1942, 30). This nature of hope consists of a reaction arising from being captivated by an anticipation. Marcel points out that this anticipation goes beyond the naturalist conviction that the future cannot be given, but only conceived. It is not important for solvability that things might not turn out as were hoped, and that is why an impasse does not stop the process of problem-solving – the problem prevails independently from the practical attempts that are made to solve it. Furthermore, hoping means not accepting the given situation, making “thematization” possible by a “domesticating of circumstances” (40).

Nevertheless, Marcel highlights that hope is no mere groundless optimism. It is always threatened by the “temptation to despair” (36) which therewith creates the field of tension out of which solvability can arise. Already at this point, it can be shown how the formalist idea of a given state and a goal state does not apply to the subjective nature of problems. Hope means to believe that there will not be an obstacle in my way, even if the experimental setup might include obstacles. Marcel sketches out this sense of “obstacle” as putting conditions in front of my hope and setting a limit to the process in which I can triumph over all disappointments. A truly hopeless person concedes altogether to the circumstances and will not have a problem anymore. Through hope, solvability is essentially connected with mineness as well. The more the problem relates to myself, the greater the incentive to hope. Consequently, between reasons for hoping which are exterior to myself and the hope for salvation, the second feature of problems emerges.

The situation I encounter is essentially problematic because it is oppressive. Oppressiveness is the root of the motivational experience of the problem. The psychological construct of motivation relates to oppressiveness as a resonance from the person, but it does not determine the problem when I am befallen by the pathos. Other than challenges or opportunities and fallacies, a problem is oppressive in an inevitable sense because it constitutes an oppression to me, i.e., it oppresses in a way that is more specifically directed against my will. It is for this connection that I pay attention to any goals that might or might not determine consecutive “goal-directed” actions of problem-solving.

Just as the impression of solvability originates from the ability to hope, to experience oppressiveness requires the ability to want. Wanting, however, is no relation to the object of will but, as various phenomenologists claim, to its value. Stein distinguishes values from “things” as the two possible contents of experience: “there are those [contents of experience; A. N. W.] ideally corresponding to experiences of content alien to the I [ichfremd], and others which are adequately grasped by an experience including the I [ichlich]. On the one side are “things,” on the other, e.g., values” (Stein, 1922, 15). Now, following Scheler, the perception of values is neither sensation in the form of my senses perceiving things nor imagination, but it is a matter of feelings, for which he adopts the term “logique du coeur” from Pascal. Scheler says: “Feeling originally directs at a proper class of objects, which are the values” (Scheler, 1916, 265).

My problem is not oppressive for me because, even though the material constellation intimidates me, its oppressiveness depends on how the values which are relevant in the situation affect me and stimulate my interest. I may experience a situation which poses no physical or social threat to me, but nevertheless causes me to have a tremendous problem, such as when I am confronted with nothing but my own consciousness. Some laboratory setups on the other hand might not be able to create actual oppressiveness because they do not carry such importance in the values of their participants. Yet, when thinking about what was expressed by the sweating and stuttering participants to the Milgram experiments (see Sharpe and Whelton, 2016), there is no reason to assume that oppressiveness is impossible to be evoked in laboratories.

Drawing on Pfänder, oppressiveness can be understood as “the feeling connected with the imagination which decides whether what is imagined is a goal or not” (Pfänder, 1900, 39). Thus, Pfänder concludes that, for something to be a goal, it neither means having certain goal-like characteristics nor aiming at a certain effect but being the object of someone’s striving. Inversely, in the case of a problem, the pulling force to this striving is the oppressiveness. The formalist idea of a goal state only appears to be valid to describe what a problem is when approaching the notion of the problem that already presupposes the practical purpose of problem-solving – a constraint which Wertheimer was willing to admit when reflecting on the validity of Gestaltist problem-solving research. Nerney draws on Duncker by calling these types of situations “problems with constructed foundations” (Nerney, 1979, 59). The concept of the goal is quite fixed, being too intellectualist to match the phenomenal quality of oppressiveness. Dreyfus, drawing on Merleau-Ponty’s critique of representationalism, emphasizes the same insight: “skillful coping does not require any representation of a goal. It can be purposive without the agent entertaining a purpose” (Dreyfus, 2004, 241). Instead of goals, the phenomenology of the problem is concerned with will and with striving.

Unlike in the case of a challenge, this oppressiveness is (more or less) urgent and dangerous for the subject. It constitutes an atmosphere within the situation that demands that the subject solve and thereby overcome it. Although novelty-seeking people might sometimes be pleased by this atmosphere, they will nonetheless strive for a solution. Atmosphere, in this context, is best understood as a “transmodal affordance” in the neo-phenomenological sense of Böhme (2001). Drawing on Böhme, Griffero (2014) takes an atmosphere to be “the specific emotional quality of a given ‘lived space”’ (37), not as a merely inward state of the subject but as an aspect of the situation itself, which is evoked by the values the situation contains. Thus, an atmosphere is an affordance because it “ecologically invites” certain meanings, especially “tertiary qualities or affective (and therefore atmospheric) ones that permeate the space in which they are perceived” (47). Ultimately, this notion of atmosphere allows the Lewinean concept of life space to emancipate itself from Gestaltist limits, complementing it with phenomenal depth.

My problem is oppressive because it makes me have certain feelings about values that are important to me. A laboratory full of computers does not achieve this experience by itself. It requires a certain atmosphere that captivates me. The inevitability of pathos appears as oppressiveness for my problem. I have the feeling that I must face this situation, for some reason in my momentary or life situation. It might be quite probable that there are occurrences that are oppressive to almost everyone, such as the outbreak of a war. To consider this notion of oppressiveness relevant is an important task for experimental design if psychologists really intend to observe problem-solving. The crucial notion of atmosphere is a connection to the third and last feature of problems.

The situation I encounter is essentially problematic because it has a problem horizon. The phenomenological notion of horizon is constitutive for perception since “the object of (transcendent) perception is characterized by its adumbrational givenness” (Zahavi, 1997, 304). It is for this form of “inner horizon” that I perceive an object because the aspects of the object which are absent to my current view are part of its totality. Thus, “in order for a perception to be a perception-of-an-object, it must be permeated by a horizontal intentionality which intends the absent profiles” (*ibid*., 305).

In addition to the “inner horizon,” there is an “outer horizon” which embeds the individual acts of experience within a holistic experience of the world. Gurwitsch summarizes: “The outer horizon comprises things at the moment not actually perceived but referred to as perceivable” (Gurwitsch, 2010, 359); and he goes on to say, “[w]ith the experience of pointing references to the outer horizon, we are at the phenomenological root and origin of the awareness we have of the world as a universal all-embracing background, context, or horizon at every moment of conscious life. Whatever material object is chosen as our theme, we perceive it within that all-embracing horizon and as pertaining to the world” (*ibid*.).

Now, when facing my problem, the horizon and background of my experiences changes due to having this problem. Most importantly, certain things become relevant because they are related to my problem. Also, my perspective on my objects of experience changes. It may be that I become more prone to detect solutions that are available on the horizon, or that I try to evade further problems. Either way, my problem’s horizon is different from my prior experience’s horizon, and it would change again if my problem turned into a challenge or a fatality.

This aspect of my experience’s horizonal intentionality facilitates understanding of what is the phenomenological core of “problematizing,” and the psychological term “problem finding” (for which see for example Getzels and Csikszentmihalyi, 1976). Whereas the experiential features of solvability and oppressiveness are implicit to the immediate experience of a problem, the state of the problem horizon can be approximated voluntarily. It does not necessarily cause the emergence of a problem but it is possible to change my perspective and my willingness to encounter a problem. The demand characteristics of psychological laboratories might be related to this possibility.

The very characteristics of the problem horizon, however, are determined by the relevance of the contents of the horizon. The notion of relevance has been elaborated by Schütz:

“[T]he articulation of the field into theme and horizon is imposed by the emergence of some unfamiliar experience, by a shift of the accent of reality from one province to another, and so on, it is characteristic of the system of intrinsic topical relevances that we may or may not direct our attention to the indications implicit in the paramount theme – indications which have the form of inner or outer horizontal structurizations or forms of topical relevances – that is, we may or may not transform these horizontal surroundings into thematic data.” (Schütz, 2011, 111).

In order to explain this idea of relevance, Schütz applies a conception of “typicality.” Experiences are recognized or chosen as typical based on a comparison with the individual stock of knowledge. This way a “system of relevance” is given: “it [sc. this system of relevance; the author] is responsible to determinate the characteristics that are selected as typical” (da Costa, 2014, 68). Thus, when I have a problem, my horizonal intentionality favors the occurrence of experiences that are relevant to the problem I face, because they appear to be typical for such a situation. Similarly, as Dreyfus and Dreyfus point out by drawing on Heidegger, “the pragmatic background plays a crucial role in determining relevance” (1988, 228). In other words, a problem’s horizon is not intellectually imposed onto one’s perception of the world, but on the contrary originates from our existential engagement with the world. Ultimately, the problem horizon is characterized by the similarity of relevant aspects among problems which exist due to their common essential features, solvability and oppressiveness.

The notion of the horizon, however, is static, whereas the experience’s dynamics in relation to the horizon should rather be identified as “perspectivity.” Graumann employs Lewin’s notion of “locomotion,” the movement in the life space, to identify the “perspectival (horizontal) structure of […] all cognitive experience” (Graumann, 1990, 8). He says: “An aspect is not a sharply bounded part of something, nor is the horizon a fixed limit, but the line of transition from the perceived to the perceivable, from the known to the knowable, from the actual to the possible, from the given to the new” (*ibid*., 9). The horizon is no passive object of experience, no surplus of the foreground, but it is originally interwoven with my experience’s perspective, and thereby constituting the problem.

Hence, *my problem happens (pathos) to me (mineness) and it demands a response while changing my experiences’ perspective, making it accessible for aspects of the horizon which are relevant for solvability and oppressiveness.* These atmospheric conditions enable problem-solving and should be considered in a psychological investigation. Ultimately, an exemplary problem can help to demonstrate the interplay of the five phenomenal features which have been introduced (namely mineness, pathos-response, solvability, oppressiveness, and the problem horizon). Yet, a written example bears the risk of suggesting certain aspects that appear to be relevant only to the way of explication. This is why it is paramount to focus on the experiential and not the linguistic qualities of the example.

Arriving early in the night at a foreign country’s airport gate, in a city I had never visited before, I had to switch terminals for transit purposes. After asking the staff for orientation, I left the building to search for a shuttle bus to reach the place from where I would board my next plane by the following morning. A taxi-driver approached me to say that the terminals would be closed at night, recommending me to take a hotel for the night. This is the moment that my problem struck me. I found myself in the middle of a tense situation that had already developed without my necessary engagement. This delay of occurrence and the retardation of my action framed a “constraint from which I could not emancipate myself.” The problem clearly was mine and this appears even more evident when thinking about the first step of my solution, which was searching for somebody with the same problem – somebody who also experienced “mineness.”

Yet, this solution and its path are no part of the very problem itself but already of the problem-solving situation. The problematic atmosphere, however, was present from the very beginning of my encountering it. Regardless of being befallen by the situation’s pathos and identified by the experience’s mineness, I did not find myself in an intractable fatality. Beyond the role of constraint, I sensed a vague impression that I would not surrender to this state, that there was a solution, rather than to ask whether there was one. This impression of hope was not directed at a goal but accompanied the oppressive feeling of imminent danger and uncertainty. I wanted my initial plans to remain in place, I wanted a secure solution, and I wanted certainty. Clearly, my perspective changed in the very moment, bringing me from a vague state of relaxation after a transatlantic flight into the very presence of a Caribbean airport. Suddenly, the strangers around me emerged in my horizon as possible sources of reassurance and knowing the time turned out to be an imminent urge. I was entirely captivated by the imminent situation.

My capacity to adapt resulted from this change of experience. In spite of this, the state did not depend on the availability of a solution but merely on the impression of a problem’s solvability. Had this feature not been present, it would have turned out to be an entirely different situation in which I had despaired instead of hoped. Whether these types of experience are possible in the laboratory, is the subsequent question.

The second step of the investigation provided evidence for a reformed understanding of problems. Instead of the functionalist formula of interrelated states, drawing on the problem as a “type of situation” allows to attend the subtler conditions to having a problem. For instance, once a subject is deprived of hope for solvability of a task, her following behavior should hardly be interpreted as problem-solving. Instead, she might be coping with a fallacy – a different type of situation. Regarding this difference will not only do justice to the phenomenal depth of problematic situations and eliminate the fallacy of taking tasks for problems, it also facilitates room for new experimental parameters of substantial predictive validity, since it suggests novel investigations of the difference of, e. g., solving a problem and taking a challenge as ways of skillful coping.

## Empirical Approaches

The field-theoretical and phenomenological considerations call for a third and final step. In order to advise a change in empirical research, it is paramount to propose concrete alternatives. The previous analyses provide rudimentary gauges for the validity of empirical approaches to investigating subjects in problematic situations. Drawing on this reference and after discussing the limits of possible empirical research, four ideas for phenomenologically sobered psychological research are presented. This way, taking the phenomenology of the problem into account, it becomes possible to assess whether there is a problem in the laboratory.

The characteristics of the laboratory situation as determined according to field-theoretical reflections relate to the five experiential features of problems. Yet, there is no simple causal relation between the laboratory setup and the subjective experiences. It is, however, possible to recognize whether or not the laboratory situation’s concept is directed at the relevant features of the problem. While it is quite clear that the mere instruction to have a problem does not take the atmospheric peculiarities into account which give rise to the experience of a problem, it is equally obvious that it remains possible to have a problem even in the artificial context of a laboratory.

The experimental situation as a region of life space is characterized by the configuration of quasi facts altered by alien influences, such as experimental instruction. Looking at the variety of experimental setups, this instruction can be an introduction, a tutorial or an affordance, in its simplest form by saying “Problem: …” But neither of these alternatives directly targets the phenomenal features of the problem. Rather, they usually do not express a systematic effort to befall the experimental subject (pathos) or to favor an experiential identification (mineness). Instead, the instruction is presented and elaborated continuously and usually does not convey the characteristic delay which is an essential part of the experience of a problem in inner time-consciousness. This means that there is a “qualitative difference in the psychological facts themselves” (Lewin) between tasks which are presented this way and authentic problems. Consequently, the actual existential relevance of the situation can only originate as a random factor from the experimental subject’s life situation, e.g., when there is some biographical relevance of the specific content, but not one stemming from experimental manipulation or instruction.

It is a curious conclusion from these circumstances that empirical research effectively depends on the extraordinary behavior shown by experimental subjects in the laboratory, such as demand characteristics. This is epistemologically comprehensible because these forms of behavior are entwined with the artificial experimental setup. In other words, the common experimental instruction resorts to forms of behavior which correlate with the laboratory situation. Without the experimenter’s efforts to evoke actual problems, research depends on the participants’ decision or habit to engage with the experimental setup. If they had a real problem, however, the problem’s oppressiveness would make them engage with the situation genuinely.

Experimental setups that do intend to evoke actual problematic experiences have to entertain different measures that actually strive to make the subjects have a problem. This is why cover stories are not sufficient for this purpose, because they seek to conceal the actual experimental hypothesis and do not contribute to creating a problematical atmosphere. Lyons’ (1970) analysis of the “hidden dialog” reveals the implicit arrangements underlying the cover story. It would be naïve to assume that the experimental manipulation depends solely on the “experimentalist’s power grab” (*ibid*., 25). Problem-solving research needs to engage with its subjects’ experimental situation in order to create problems.

Furthermore, adopting commonly reported everyday problems does not serve this purpose, either, since the laboratory, as shown by field theory, is actually an entirely different form of life space. “On an island quite isolated from the life of society,” in Lewin’s picturesque phrasing, different things become relevant. Despite everything else, there are four (exemplary) directions of empirical research that carry the possibility of augmenting empirical designs so that actual problem-solving research becomes possible. Ultimately and in general, the empirical questions which arise in the light of the phenomenology of the problem invite one to reflect on empirical methodology.

Among the considerable alternative methodological approaches, the first one is straightforwardly to say that there are already experimental paradigms, such as ethnomethodology, which indirectly include the intention to cause a problem by perforating or defying the participants’ routine. In ethnomethodology’s technique of the “breaching experiments” (on which see Kew, 1986), puzzling and disorganized situations are provoked which occasionally succeed to create pathos. Following the phenomenology of the problem, this might not be enough to evoke the experience of an actual problem, but indicates by way of an example fruitful resources which should be used in psychology. Yet there is no engagement of the discipline with ethnomethodology as of today. This example also illustrates that spirited and creative experimental design guided by phenomenologically valid purposes can contribute to amplify the empirical validity of laboratory research.

A second methodological approach is the application of unobtrusive forms of observation. In their approach this application avoids the effects of instruction. A recent development of media psychology hints that there is remarkable potential for this form of research using digital data sources, such as live streaming. live streaming is a client-created form of content on video hosting websites which transmit content in real-time (for a detailed investigation of live streaming’s feasibility for empirical psychology, see Wendt, 2017b). It is remarkable to what extent these broadcasts resemble laboratory setups based on video capture, for example in usability research. Another striking similarity is given between the traditional contents of problem-solving research, since live streaming primarily hosts video gaming. Furthermore, the data collected by this method matches with the concept of Natural Occurring Data Sets (NODS) and Big Data (Goldstone and Lupyan, 2016).

An example can help to elucidate these potentials given to research employing live streaming. As Graumann (1969) points out, problematic situations are closely linked to the experience of frustration. Phenomenologically speaking, this observation is entwined with what has been discussed as the origin of solvability in hope. More precisely, in the experience of frustration, hope for the problem’s solvability fades. However, really frustrating experiences, which are characterized by manifold emotional reactions and their expressions identified in the phenomenology of feelings, even if they are folk-psychologically unsuspected, such as joy (see Elpidorou, 2017), can rarely be encountered in the laboratory. This seems partly because these experiences and reactions are not investigated, due to the predominance of intellectualist theory in psychology, and partly because the laboratory setup restricts the natural sparking of such experiences. In live streaming, on the other hand, subjects freely experience and express frustration. It is reasonable to assume that the causes for it are related to the setting where streamers might perceive an incentive to express themselves intuitively in view of voluntary exposure to an audience, and the nature of their interaction is often in situations that are frustrating by nature, such as difficult or unfair games.

It is arguably possible to manipulate frustrating conditions in the laboratory as well, namely when something is frustrating in the etymological sense of the word (Lat. *fraus* – harm): Not only may a person be frustrated but, seen from a situational standpoint, their frustration may primordially emerge from harm being inflicted upon their present state, e.g., by the instruction to solve a task which actually does not have a material solution (consequently, the natural expectation to perform achievable tasks is harmed). But even when setting an unsolvable task, it remains unlikely that one will observe in a laboratory what can be seen easily and frequently in a live stream: subjects shout, quit, or even get violent under eventually standardized observation conditions – occurrences which would mean the end to most contemporary laboratory investigation. Despite overt limitations to the interpretability of live streaming as a data source, these brief considerations of a handy example succeed in demonstrating the scope of the situational setup when investigating problems. In live streaming, courses of action unfold which are highly unlikely or institutionally impossible to manufacture in the same way in a laboratory.

Nevertheless, there are two methodological concerns here. On the one hand, it is not safe to say that all laboratory effects, such as demand characteristics, only occur in the laboratory. Sociology seems to favor the idea that there are further conditions to this biased behavior. Cooley’s (1902) concept of the looking-glass-self highlights the anthropological dimension of such effects that seem to originate in the most elementary act of self-perception. On the other hand, the similarity with traditional problem-solving tasks does not provide a sufficient conceptual foundation to consider the phenomenology of the problem. Since the subject matter of the standardized and most accessible live streams are video games, research on live streaming also requires the implementation of game theory in order to explore the depth of these gaming experiences.

Third, there are possibilities to enhance the experimental setup, namely by making use of forms of communication which comprise atmospheric complexity. Certainly, this is no easy task. On the contrary, it requires a sort of additional mastery from the experimenter. However, there are several ways to approach this matter which do not all include the necessity of active creative production in laboratory setups. One favorable way to increase the credibility and impact of experimental instructions is indirectly hinted at in one of Lewin’s thoughts:

“The most complete and concrete descriptions of situations are those which writers such as Dostoevski have given us. These descriptions have attained what the statistical characterizations have most notably lacked, namely, a picture that shows in a definite way how the different facts in an individual’s environment are related to each other and to the individual himself. The whole situation is presented with its specific structure. This means that the single factors of the situation are not given as characteristics which can be arbitrarily combined in a ‘summative’ way.” (Lewin, 1936, 13).

Empirical research has long rejected the implementation of artistic production into experimental design out of a danger of conflating itself with folk psychology. Yet, the controlled and directed use of well-investigated effects of such descriptions should suit with even the concepts of cognitivist operationalism. Another concern might be the lack of control that this type of investigation would seem to imply, but this apparent control has always been founded on a reductionist notion of language that disregards the complexity of natural languages. No instruction whatsoever can be liberated of this effect.

The fourth approach for phenomenologically sobered empirical research is the pending rejuvenation of ecological psychology. In the second half of the 20th century, there have been several attempts to investigate the peculiarities of the situated subject. While the relation between system and environment was favored in behaviorist and cognitivist research, phenomenology has explored the notion and experience of the situation (Schott, 1991). The research into this field might have been neglected in the 21st century due to the recent preference for quantitative methods, but in the current spring of mixed-method approaches, it is worth reconsidering these traditions of thought as viable once more.

Ultimately, these methodological considerations have to remain no more than proposals without a decisive criterion for their implementation. Such a criterion, however, is a matter of methodological discussion in the empirical sciences which principally involves the question of introspection. By not giving more than a prospective glance to necessary deliberation, it can be said that it depends on the relevance of subjective experience in psychology whether or not the phenomenology of the problem will be considered meaningful in the discipline. In spite of this, the prevalent customs of discourse among psychologists do not invite confidence about the probability of this change of view.

The orthodox form of Husserlian phenomenology itself has dismissed the connection to introspectionism and descriptive psychology, on the grounds that both entail an empirical notion which does not provide insight into the transcendental constitution of consciousness. However, less rigorous approaches within the phenomenological movement have previously considered the connection between both domains. The exploration of pre-reflective experience calls for some empirical correlate which might be encountered in an introspection unlike the classical highly language-focused attempts, such as in the group of Würzburg psychologists around Külpe. The investigation of states as subtle as atmospheres could be a most promising direction for this research to head in. In light of this consideration, the matter of the problem emerges at the very center of contemporary discourse about introspection. As an example of the recent relevance of this debate, Petitmengin and Bitbol (2009) adopted a Brentanian view to defend introspection. Their practical considerations resemble the ideas of the Würzburg-based psychologists insofar as they recommend the education of specialists of introspection.

Regardless of this, investigation into the relation between introspection and the phenomenology of the problem calls for future reflection. Another important matter at stake here is the discriminative exploration of the problem. Once the ubiquitous credo “all life is problem-solving” has been defied, the question should be raised as to what situational alternatives do populate the life space. Challenges, fallacies, and opportunities are considerable modes of the situation and their examination might bear noteworthy potential – especially for problem-solving research. A different approach can be to draw on Fales’ phenomenology of the question. He separates four principal categories of questions: “Questions are either a matter of knowledge, or of belief, or of taste, or of action” (Fales, 1943, 60). These different categories of the question can be used to distinguish different types of situations, such as problems. However, based on a more complex notion of the situation, psychological investigations will succeed in discriminating the experience of a person attending a task, facing a challenge, or solving a problem, up to the point of making valid predictions about their consecutive behavior. For passionate experimental psychologists, it might turn out to be a problem that laboratories rarely foster the experience of problems for the experimental subjects. The common types of situations which are created in order to investigate problem-solving are mere tasks and do not let the experiential features of authentic problems occur. Far from being a catastrophe and certainly being more than a challenge, these circumstances should inspire more creative approaches to carrying out experimental psychology. In order to inspire advances in experimental methodology, the following step must be to establish a continuous, constructive and reciprocal dialog between empirical and phenomenological study and discourse.

## Author Contributions

The author confirms being the sole contributor of this work and has approved it for publication.

## Conflict of Interest Statement

The author declares that the research was conducted in the absence of any commercial or financial relationships that could be construed as a potential conflict of interest.

## References

[B1] Anderson C. A. Lindsay J. J. Bushman B. J. (1999). Research in the psychological laboratory: truth or triviality? *Curr. Dir. Psychol. Sci.* 8 3–9. 10.1111/1467-8721.00002

[B2] Anderson J. R. (1993). *Rules of the Mind.* Hillsdale, NJ: Lawrence Erlbaum.

[B3] Asch S. E. (1951). “Effects of group pressure on the modification and distortion of judgments,” in *Groups, Leadership and Men*, ed. Guetzkow H. (Pittsburgh, PA: Carnegie Press), 177–190.

[B4] Böhme G. (2001). *Aisthetik: Vorlesungen über Ästhetik als allgemeine Wahrnehmungslehre.* München: Fink.

[B5] Braem S. Liefooghe B. De Houwer J. Brass M. Abrahamse E. L. (2017). There are limits to the effects of task instructions: making the automatic effects of task instructions context-specific takes practice. *J. Exp. Psychol* 43 394–403. 10.1037/xlm0000310 27617776

[B6] Cooley C. H. (1902). *Human Nature and the Social Order.* New York, NY: C. Scribner’s sons.

[B7] Crowne D. P. Marlowe D. (1960). A new scale of social desirability independent of psychopathology. *J. Consult. Psychol.* 24 349–354. 10.1037/h0047358 13813058

[B8] da Costa T. (2014). Between relevance systems and typification structures: alfred schutz on habitual possessions. *Phenomenol. Mind* 66–72.

[B9] Di Mascio R. Kalyuga S. Sweller J. (2016). The effect of wording and placement of task instructions on problem-solving creativity. *J. Creat. Behav.* 1–19. 10.1002/jocb.157

[B10] Dörner D. Funke J. (2017). Complex problem solving: What It Is and What It Is Not. *Front. Psychol.* 8:1153. 10.3389/fpsyg.2017.01153 28744242 PMC5504467

[B11] Dreyfus H. (1972). *What Computer Can’t Do: A Critique of Artificial Reason.* Cambridge, MA: MIT Press.

[B12] Dreyfus H. (2004). “Merleau-Ponty and recent cognitive science,” in *Skillful Coping: Essays on the Phenomenology of Everyday Perception and action*, eds Dreyfus H. L. Wrathall M. A. (Oxford: Oxford University Press), 231–248. 10.1017/CCOL0521809894.006

[B13] Dreyfus H. Dreyfus S. (1988). “Making a mind versus modeling the brain,” in *Skillful Coping: Essays on the Phenomenology of Everyday Perception and Action*, eds Dreyfus H. L. Wrathall M. A. (Oxford: Oxford University Press), 205–230. 10.1016/j.bpsc.2017.04.007

[B14] Elpidorou A. (2017). Emotions in early sartre: the primacy of frustration. *Midwest Stud. Philos.* 41 241–259. 10.1111/misp.12075

[B15] Fales W. (1943). Phenomenology of questions. *Philos. Phenomenol. Res.* 4 60–75. 10.2307/2103005

[B16] Funke J. (2014). “Problem solving: What are the important questions?,” in *Proceedings of the 36th Annual Conference of the Cognitive Science Society*, eds Bello P. Guarini M. McShane M. Scassellati B. (Austin, TX: Cognitive Science Society), 493–498.

[B17] Getzels J. W. (1982). “The problem of the problem,” in *New Directions for Methodology of Social and Behavioral Science: Question Framing and Response Consistency* Vol. 11 ed. Hogarth H. (San Francisco, CA: Jossey Bass), 37–49.

[B18] Getzels J. W. Csikszentmihalyi M. (1976). *The Creative Vision: A Longitudinal Study of Problem Finding in Art.* New York, NY: John Wiley & Sons.

[B19] Goldstein K. (1934). *Der Aufbau des Organismus. Einführung in die Biologie unter besonderer Berücksichtigung der Erfahrungen am kranken Menschen.* Haag: Nijhoff 10.1007/978-94-017-7141-2

[B20] Goldstone R. L. Lupyan G. (2016). Discovering psychological principles by mining naturally occurring data sets. *Topics Cognit. Sci.* 8 548–568. 10.1111/tops.12212 27404718

[B21] Goldstone R. L. Pizlo Z. (2009). New perspectives on human problem solving. *J. Probl. Solving* 2 1–5. 10.7771/1932-6246.1055

[B22] Graumann C. F. (1969). *Motivation.* Bern-Stuttgart: Akademische Verlagsgesellschaft.

[B23] Graumann C. F. (1990). “Perspectival structure and dynamics in dialogues,” in *The Dynamics of Dialogue*, eds Markova I. Foppa K. (New York, NY: Springer), 105-127.

[B24] Griffero T. (2014). Atmospheres and lived space. *Stud. Phaenomenol.* 14 29–51.

[B25] Guillot M. (2017). I me mine: on a confusion concerning the subjective character of experience. *Rev. Philos. Psychol.* 8 23–53. 10.1007/s13164-016-0313-4 28386303 PMC5362670

[B26] Gurwitsch A. (1949). Gelb-goldstein’s concept of “concrete” and “categorial” attitude and the phenomenology of ideation. *Philos. Phenomenol. Res.* 10 172–196. 10.2307/2104073

[B27] Gurwitsch A. (2010). “The thematic field,” in *The Collected Works of Aron Gurwitsch (1901-1973)*, ed. Zaner R. (Dordrecht: Springer), 301–365. 10.1007/978-90-481-3346-8_10

[B28] Husserl E. (1917). “Phänomenologie und Psychologie,” in *Freiheit und Gnade*, ed. Stein E. (Freiburg: Herder), 195–230.

[B29] Hutto D. D. (2008). Articulating and understanding the phenomenological manifesto. *Abstracta* 4 10–19.

[B30] Kew F. (1986). Playing the game: an ethnomethodological perspective. *Int. Rev. Sociol. Sport* 21 305–322. 10.1111/tops.12234 27901311

[B31] Laird J. E. Newell A. Rosenbloom P. S. (1987). Soar: an architecture for general intelligence. *Artif. Intell.* 33 1–64. 10.1016/0004-3702(87)90050-6 1643846

[B32] Landsberger H. A. (1958). *Hawthorne Revisited.* Ithaca, NY: Cornell University.

[B33] Langley P. Rogers S. (2005). “An extended theory of human problem solving,” in *Proceedings of the twenty-seventh annual meeting of the cognitive science society*, Stresa, 27.

[B34] Langley P. Trivedi N. (2013). Elaborations on a theory of human problem solving. *Adv. Cognit. Syst.* 3 1–12.

[B35] Levinas E. (1986). “The Trace of the Other,” in *Deconstruction in Context*, ed. Taylor M. (Chicago: University of Chicago Press), 345–359.

[B36] Lewin K. (1927). Gesetz und Experiment in der Psychologie. *Symposium* 1 375–421.

[B37] Lewin K. (1936). *Principles of Topological Psychology.* New York, NY: McGraw-Hill Book Company 10.1037/10019-000

[B38] Lewin K. (1939). Field theory and experiment in social psychology: concepts and methods. *Am. J. Soc.* 44 868–896. 10.1086/218177

[B39] Lewin K. (1940). “Formalization and progress in psychology,” in *Field Theory in Social Sciences*, ed. Lewin K. (New York, NY: Harper & Brothers).

[B40] Lewin K. (1944). “Constructs in psychology and psychological ecology,” in *Field Theory in Social Sciences*, ed. Lewin K. (New York, NY: Harper & Brothers).

[B41] Lyons J. (1970). The hidden dialogue in experimental research. *J. Phenomenol. Psychol.* 1 19–29. 10.1186/1752-0509-8-13 24507381 PMC3927870

[B42] Marcel G. (1942). “Sketch of a phenomenology and a metaphysic of hope,” in *Homo viator. Introduction to a Metaphysic of Hope*, ed. Marcel G. (Indiana: St. Augustine’s Press).

[B43] Nerney G. (1979). The gestalt of problem-solving: an interpretation of max wertheimer’s “productive thinking”. *J. Phenomenol. Psychol.* 10 56–80. 10.1163/156916279X00059

[B44] Newell A. Simon H. A. (1972). *Human Problem Solving.* Englewood Cliffs, NJ: Prentice-Hall.

[B45] Ohlsson S. (2012). The problems with problem solving: reflections on the rise, current status, and possible future of a cognitive research paradigm. *J. Probl. Solving* 5 101–128. 10.7771/1932-6246.1144

[B46] Orne M. T. (1962). On the social psychology of the psychological experiment: with particular reference to demand characteristics and their implications. *Am. Psychol.* 17 776–783. 10.1037/h0043424

[B47] Petitmengin C. Bitbol M. (2009). Listening from within. *J. Conscious. Stud.* 16 363–404.

[B48] Pfänder A. (1900). *Phänomenologie des Wollens. Eine psychologische Analyse.* Leipzig: Johann Ambrosius Barth.

[B49] Popper K. R. (1999). *All Life is Problem Solving.* Hove: Psychology Press.

[B50] Quesada J. Kintsch W. Gomez E. (2005). Complex problem-solving: a field in search of a definition? *Theor. Issues Ergonomics Sci.* 6 5–33. 10.1080/14639220512331311553

[B51] Rosenthal R. Rubin D. B. (1978). Interpersonal expectancy effects: the first 345 studies. *Behav. Brain Sci.* 1 377–386. 10.1017/S0140525X00075506

[B52] Sartre J.-P. (1956). *Being and Nothingness.* New York, NY: Washington Square Press.

[B53] Scheler M. (1916). *Der Formalismus in der Ethik und die materiale Wertethik.* Halle: Max Niemeyer.

[B54] Schott E. (1991). *Psychologie der Situation. Humanwissenschaftliche Vergleiche.* Heidelberg: Asanger.

[B55] Schütz A. (2011). Reflections on the problem of relevance, in: collected papers V: phenomenology and the social sciences. *Hague* 3 93–199.

[B56] Sharpe D. Whelton W. J. (2016). Frightened by an old scarecrow: the remarkable resilience of demand characteristics. *Rev. Gen. Psychol.* 20 349–368. 10.1037/gpr0000087

[B57] Stein E. (1922). Beiträge zur philosophischen Begründung der Psychologie und der Geisteswissenschaften. *Jahrbuch Philos. phänomenol. Forschung* 5 1–283.

[B58] Thompson E. (2007). *Mind in Life. Biology, Phenomenology, and the Sciences of Mind. Cambridge.* London: Harvard University Press.

[B59] Tversky A. Kahneman D. (1981). The framing of decisions and the psychology of choice. *Science* 211 453–458. 10.1126/science.7455683 7455683

[B60] Vaihinger H. (1965). *The Philosophy of “As If ”: A System of the Theoretical, Practical, and Religious Fictions of Mankind.* London: Routledge and Kegan Paul.

[B61] Waldenfels B. (1994). Response und Responsivität in der Psychologie. *J. Psychol.* 2 71–80.

[B62] Waldenfels B. (2015). *Sozialität und Alterität: Modi sozialer Erfahrung.* Frankfurt am Main: Suhrkamp Verlag.

[B63] Weisberg R. W. (2015). Toward an integrated theory of insight in problem solving. *Thinking & Reasoning* 21 5–39. 10.1080/13546783.2014.886625

[B64] Wendt A. N. (2017a). On the benefit of a phenomenological revision of problem solving. *J. Phenomenol. Psychol.* 48 240–258. 10.1163/15691624-12341330

[B65] Wendt A. N. (2017b). The empirical potential of Live Streaming beyond cognitive psychology. *J. Dynamic Decis. Mak.* 3 1–9.

[B66] Yoshimi J. (2017). The phenomenology of problem solving. *Grazer Philosophische Stud.* 94 391–409. 10.1163/18756735-09403006

[B67] Zahavi D. (1997). Horizontal intentionality and transcendental intersubjectivity. *Tijdschrift voor Filosofie* 59 304–321.

